# Conditional knock out of N-WASP in keratinocytes causes skin barrier defects and atopic dermatitis-like inflammation

**DOI:** 10.1038/s41598-017-07125-8

**Published:** 2017-08-04

**Authors:** Pazhanichamy Kalailingam, Hui Bing Tan, Neeraj Jain, Ming Keat Sng, Jeremy Soon Kiat Chan, Nguan Soon Tan, Thirumaran Thanabalu

**Affiliations:** 1 School of Biological Sciences, Nanyang Technological University, 60 Nanyang Drive, Singapore, 637551 Republic of Singapore; 2 Lee Kong Chian School of Medicine, Nanyang Technological University, 59 Nanyang Avenue, Singapore, 636921 Republic of Singapore; 3 Institute of Molecular and Cell Biology, 61 Biopolis Drive, Singapore, 138673 Republic of Singapore; 4 KK Women’s and Children’s Hospital, 100 Bukit Timah Road, Singapore, 229899 Republic of Singapore

## Abstract

Neural-Wiskott Aldrich Syndrome Protein (N-WASP) is expressed ubiquitously and regulates actin cytoskeleton remodeling. In order to characterize the role of N-WASP in epidermal homeostasis and cutaneous biology, we generated conditional N-WASP knockout mouse using CK14-cre (cytokeratin 14) to ablate expression of N-WASP in keratinocytes. N-WASP^K14KO^ (*N-WASP* ^*fl/fl*^ *; CK14-Cre*) mice were born following Mendelian genetics suggesting that N-WASP expression in keratinocytes is not essential during embryogenesis. N-WASP^K14KO^ mice exhibited stunted growth, alopecia, dry and wrinkled skin. The dry skin in N-WASP^K14KO^ mice is probably due to increased transepidermal water loss (TEWL) caused by barrier function defects as revealed by dye penetration assay. N-WASP^K14KO^ mice developed spontaneous inflammation in the neck and face 10 weeks after birth. Histological staining revealed thickening of the epidermis, abnormal cornified layer and extensive infiltration of immune cells (mast cells, eosinophils and T-lymphocytes) in N-WASP^K14KO^ mice skin compared to control mice. N-WASP^K14KO^ mice had higher serum levels of IL-1α, TNF-α, IL-6 and IL-17 compared to control mice. Thus our results suggest that conditional N-WASP knockout in keratinocytes leads to compromised skin barrier, higher infiltration of immune cells and hyperproliferation of keratinocytes due to increased production of cytokines highlighting the importance of N-WASP in maintaining the skin homeostasis.

## Introduction

Actin cytoskeleton remodeling and actin polymerization play critical roles in cell motility, cell growth, phagocytosis, cell polarity and other physiological processes^1^. Temporal and spatial regulation of actin polymerization are regulated by actin nucleation factors such as the Arp2/3 complex^2^. The activity of the Arp2/3 complex is, in turn, regulated by nucleation promoting factors such as N-WASP, which is a member of the WASP family of proteins^3^. N-WASP consists of an N-terminal WASP homology domain (WH1), a central GTPase-binding domain (GBD), proline-rich region (PRR), and a C-terminal Verprolin Central Acidic (VCA) domain^4^. N-WASP has been shown to adopt a closed conformation that is stabilized by the binding of WIP (WASP Interacting Protein) to the WH1 domain^5, 6^ and binding of activated Cdc42 to the GBD domain relieves the auto-inhibition of N-WASP^7^. The closed conformation of N-WASP can also be relieved by the binding of SH3-containing proteins such as GRB2 to the PRR^8^ and the open conformation can be stabilized by the phosphorylation of Tyrosine256 (Tyr253 in mouse) by focal adhesion kinase and Src family kinases^9^. N-WASP plays an essential role in many cellular processes such as invadopodium formation associated with breast cancer invasion and lung metastasis^10, 11^. Epithelial cells in epithelia are connected to each other through 4 types of junctions; tight junctions, adherens junctions, gap junctions and desmosomes^12^. Tight junctions, consisting of networks of claudins and other proteins, are found near the apical surface and serve as intercellular seals between adjacent epithelial cells^13^. Adherens junctions are formed through the interaction of cadherins (E-cadherin in epithelia) presented on adjacent cells and are strengthened by the interaction of the cadherin cytoplasmic tail with the actin cytoskeleton^14^. Gap junctions formed through connexins and other proteins provide intercellular channels for passage of ions and small molecules between cells^15^. Desmosomes are also formed through the interaction of cadherins from adjacent cells but are strengthened by the interaction of cadherin cytoplasmic tail with intermediate filaments such as keratin^16^. Adherens junctions play an important role in the formation of tight junction and desmosomes^17^. N-WASP plays a critical role in the maintenance of the epithelial junctional actin cytoskeleton through a non-canonical post-nucleation pathway^18^. N-WASP has crucial role in cell adhesion and spreading^19^, as well as lung^10^ and colorectal metastasis^20^.

Skin, the largest organ in the body, is divided into two distinct layers, dermis and epidermis. The keratinocyte forms a stratified continuous epithelial layer over the surface of the dermal membrane to form the epidermal layer. Fibroblasts are responsible for the formation of connective tissues through the secretion of collagen and are the major cell types found in the dermis^21^. The basal layer of the epidermis is populated with proliferative cells, which divide and differentiate to form the spinous layer, granular layer and stratum corneum (SC). Keratinocytes in the granular layer are connected by tight junctions, adherens junctions, and desmosomes^22^. The SC are predominantly made up of enucleated keratinocytes, known as corneocytes that are closely connected by corneodesmosomes and tight junctions to form the skin barrier. The skin serves as a protective layer for the animal, preventing tissue dehydration, exposure to external allergens and UV radiation^23^.

A defective skin barrier leads to skin disorders, including atopic dermatitis (AD), eczema, and allergy^24^. A defective skin barrier leads to excessive water loss and also allows pathogens or allergens to penetrate into the epidermis, causing the subsequent dysregulated release of proinflammatory cytokines and chemokines^25^. Both Th-1 and Th-2 cell responses are critical for the pathogenesis of atopic dermatitis as they produce cytokines which regulate the inflammatory response in AD^26^. Besides, mast cells and eosinophils, serum IgE levels are also increased in atopic dermatitis mouse models. All these immune cells and cytokines together produce an immune response leading to cutaneous inflammation of the skin^27^. In order to study the role of N-WASP in skin biology, two research groups have generated mice with conditional knockout of N-WASP in keratinocytes using CK5-cre but with contrasting results, either with normal keratinocyte proliferation^28^ or with epidermal thickening due to keratinocyte hyperproliferation^29^.

We have generated mice in which the expression of N-WASP was ablated in the keratinocytes using CK14-cre as the driver. The mice with the expected genotype (*N-WASP* ^*fl/fl*^ *; K14-Cre*), N-WASP^K14KO^ were born at the expected Mendelian ratio. The N-WASP^K14KO^ mice displayed many characteristics of atopic dermatitis, including compromised skin barrier, abnormal cornification and spontaneous inflammation around the face and neck associated with increased infiltration of immune cells and hair loss. In summary, our results suggest that N-WASP plays a critical in post-natal skin homeostasis.

## Results

### Conditional knockout of N-WASP in skin epidermis leads to dry, wrinkled and scaly skin

**Figure 1:**
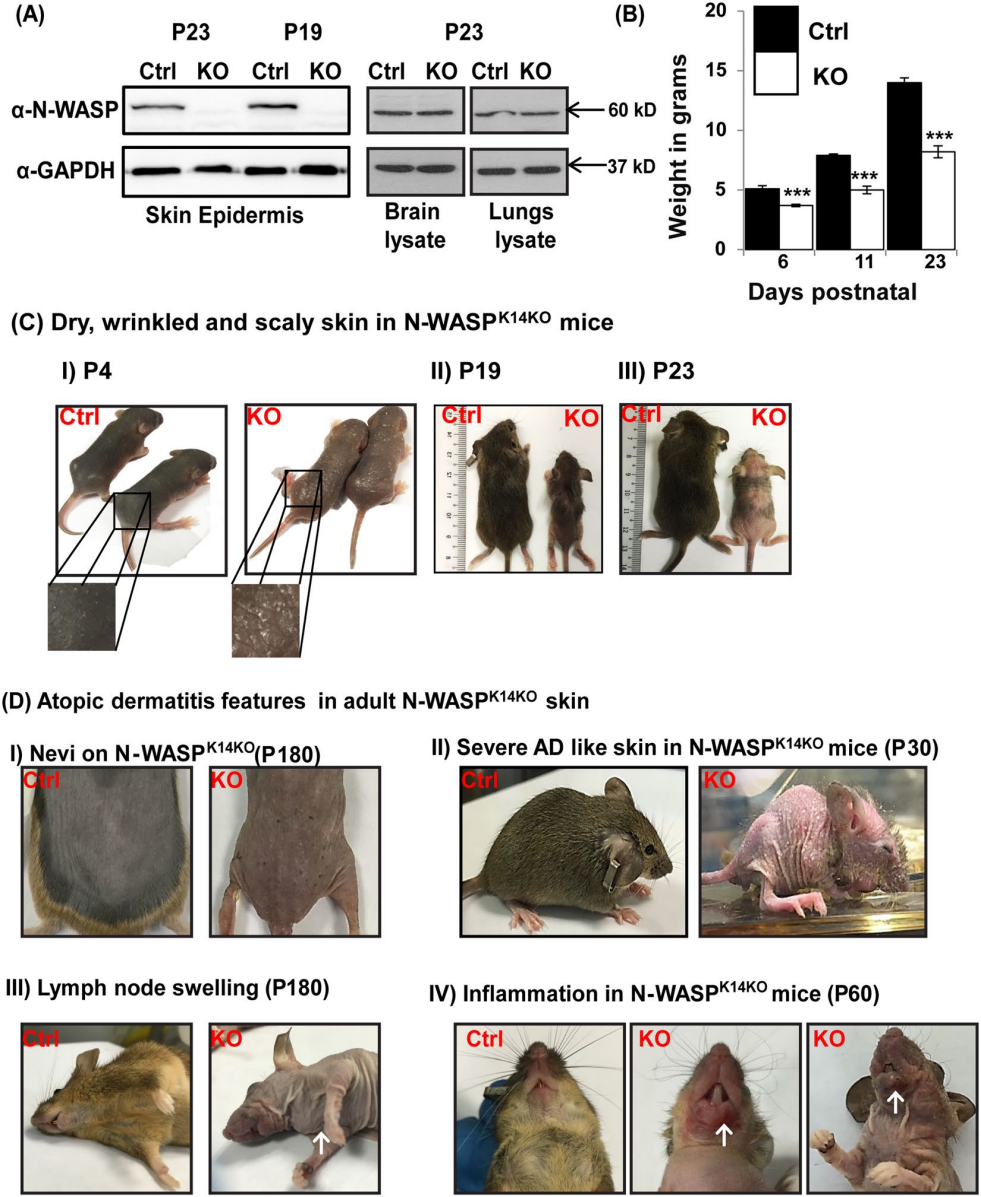
N-WASP knockout in keratinocytes leads to dry skin. **(A)** Western blot analysis for N-WASP expression in skin epidermis (P19 and P23), brain and lung from P23 mice (n = 3) (N-WASP^K14KO^ and N-WASP^fl/fl^), (Blots were cropped and full length western blot images are in supplementary Fig. S6A), **(B)** Average body weight of represented mice, (n = 5) **(C)** Dry, wrinkled and scaly skin in N-WASP^K14KO^ mice; Skin from control and N-WASP knockout mice on P4, (CI), P19 (CII) and P23 (CIII) (n = 20). **(D)** Atopic dermatitis features in adult N-WASP^K14KO^ skin; Nevi on skin (DI), severe atopic like skin (DII), (↑) indicates lymph node swelling (DIII), and (↑) indicates inflammation (DIV) (n = 20) in N-WASP^K14KO^ mice skin. Results are mean ± SEM ***p < 0.001.

In order to study the role of N-WASP in the epidermis, we generated keratinocyte-specific N-WASP knockout mice by crossing *N-WASP* ^*fl/fl*^ mice with K14-cre mice. The heterozygous *N-WASP* ^*fl/WT*^ *K14-cre* mice obtained in the F_1_ generation (Fig. S1A) were crossed with *N-WASP* ^*fl/fl*^ to generate *N-WASP* ^*fl/fl*^; *K14-cre* (N-WASP^K14KO^), conditional keratinocyte N-WASP knockout mice. Twenty-five percent of offspring were N-WASP^K14KO^, consistent with the expected Mendelian ratio (Fig. S1B). PCR with tail genomic DNA from N-WASP^K14KO^ mice revealed the excision of exons 3 and 4 of the N-WASP gene (Fig. S1C). We verified conditional knockout of N-WASP in the epidermis by carrying out western blot analysis of protein extracted from the epidermis of N-WASP^K14KO^ and N-WASP^fl/fl^ (Ctrl) mice. The epidermal protein lysate collected from N-WASP^K14KO^ and control mice showed the lack of N-WASP expression at postnatal day 19 (P19) (Fig. 1A) and 23 (P23) (Fig. 1A) (n = 3). Expression of N-WASP was not affected in brain or lung lysate isolated from P23 N-WASP^K14KO^ mice (Fig. 1A). These results suggest that N-WASP knockout is highly specific to epidermal keratinocytes. Both P19 (Telogen phase) and P23 (Anagen phase) N-WASP^K14KO^ mice exhibited alopecia, barrier defects and epidermal hyperplasia. We used P23 N-WASP^K14KO^ mice for all our subsequent studies.

N-WASP^K14KO^ mice exhibited growth defects as determined by body weight (n = 5) (Fig. 1B), size, external morphology and hair cycle (Fig. 1C-II and C-III). The reduced body weight could be due to esophageal defects (wide lumen size and abnormal mucosa) (n = 5, 60% of N-WASP^K14KO^ mice had gross defect, Fig. S1D) and/or increased epidermal water loss^30, 31^. Dry, wrinkled and scaly skin (Fig. 1C-I) were observed in N-WASP^K14KO^ mice (n = 20) during P1 to P7 days (postnatal), and inflammation was observed in the neck and face (P60 days) (Fig. 1D-IV), and nevi (P180 days) (Fig. 1D-I) appeared in the dorsal skin. Moreover, severe alopecia was observed in N-WASP^K14KO^ mice after P180 days (85%, n = 17) (Fig. 1D-I). Lymph node swelling was also observed in N-WASP^K14KO^ mice (Fig. 1D-III). We also found that 10% of N-WASP^K14KO^ mice developed severe atopic dermatitis-like skin and died before the age of 30 days (Fig. 1D-II). *Staphylococcus aureus* has been shown colonize the skin of AD patients^32^. Thus we swabbed the ear skin of mice (P23, N-WASP^K14KO^ and control mice) and cultured the bacteria on mannitol agar plates to quantify the *S. aureus* colonization of the mice. The data revealed a significant increase in the number of bacterial colonies from skin swab of N-WASP^K14KO^ mice compared to skin swab from control mice (Fig. S1E). The yellow colonies on the plates were confirmed as *S. aureus* by PCR (Fig. S1F) amplification of *Staphylococcal* enterotoxin B (SEB). These results suggest that N-WASP^K14KO^ mice developed an atopic dermatitis-like phenotype and had increased colonization by *S. aureus* bacteria.

### Loss of N-WASP expression in keratinocytes causes epidermal hyperplasia in N-WASP^K14KO^ mice

**Figure 2:**
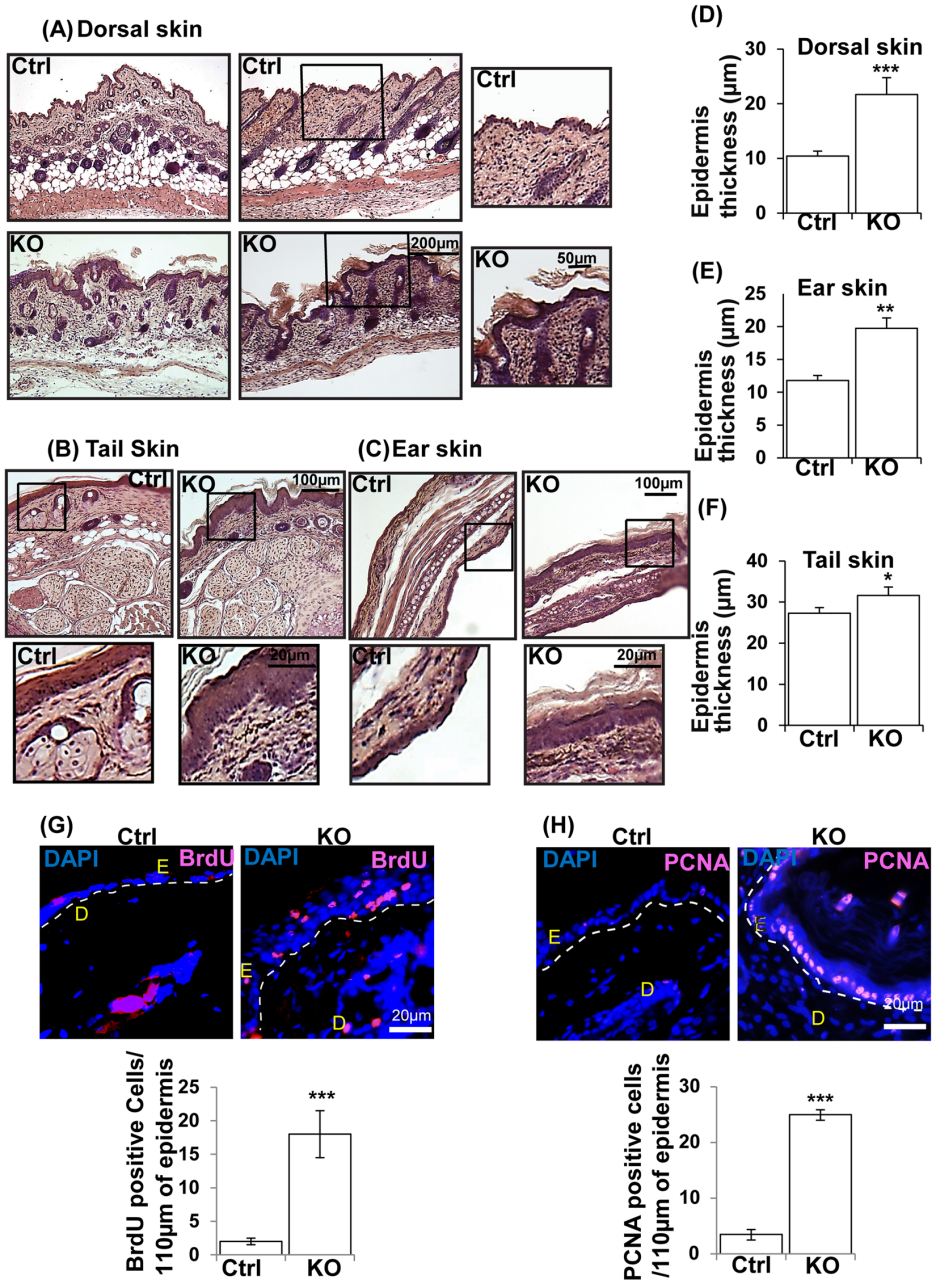
Loss of N-WASP expression in keratinocytes caused hyperproliferation of keratinocytes in N-WASP^K14KO^ mice. **(A)** H & E stained dorsal skin, tail **(B)** and ear **(C)** sections of N-WASP^K14KO^ and control mice on 23^rd^ day (n = 10). Quantification of epidermal thickening in skin **(D)**, ear **(E)** and tail **(F)** of control mice and N-WASP^K14KO^ mouse skin sections (n = 5). **(G)** BrdU staining: Quantification of BrdU positive cells in epidermis of control (n = 3) and N-WASP^K14KO^ (n = 4) mice skin at P23 **(H)** PCNA immunostaining of paraffin embedded dorsal skin sections of control and N-WASP^K14KO^ mice at P23 and quantification of PCNA positive cells (n = 3). Results are mean ± SEM ***p < 0.001, **p < 0.01, *p < 0.05.

In order to characterize the role of N-WASP in the epidermis, haematoxylin and eosin (H&E) and immunofluorescence (IF) staining were carried out in N-WASP^K14KO^ and control P19 (n = 6) and P23 (n = 10) mice. H&E staining on P19 and P23 mice revealed that the epidermis was significantly thicker in N-WASP^K14KO^ compared to N-WASP^fl/fl^ (control) and N-WASP^fl/WT^; K14-Cre mice (Fig. 2A, S2A) and similar thickening of the epidermis in N-WASP^K14KO^ was found in the tail (Fig. 2B, S2B) and ear (Fig. 2C, S2C). Quantification of epidermal layer thickness in dorsal skin (Fig. 2D, S2D), ear (Fig. 2E, S2E) and tail (Fig. 2F, S2F) showed a significant increase in N-WASP^K14KO^ animals compared to control mice on P19 (n = 6) and P23 (n = 5). To investigate if the epidermal thickening in N-WASP^K14KO^ mice is due to increased proliferation, differentiation or both, we assessed BrdU incorporation and immunostaining for proliferation and differentiation markers. We carried out immunostaining with anti-BrdU antibodies on skin sections of P23, N-WASP^K14KO^ and control mice injected with BrdU. The number of BrdU positive cells in epidermis was found to be increased in N-WASP^K14KO^ mice (n = 4) compared to controls (n = 4) suggesting increased keratinocyte proliferation in N-WASP^K14KO^ mice (Fig. 2G). IF staining was also carried out for PCNA in N-WASP^K14KO^ and control mice (P19 and P23) (Fig. 2H, S2G). PCNA positive cells in the basal layer were quantified and the data revealed that N-WASP^K14KO^ mice had a significantly higher (23 ± 5, P23 days) number of PCNA positive keratinocytes compared to control mice (3 ± 5, P23 days) (Fig. 2H) which was further confirmed by western blot analysis of PCNA expression (Fig. S3E). These data suggest that N-WASP^K14KO^ mice display increased keratinocytes proliferation resulting in epidermal hyperplasia.

To characterize keratinocyte differentiation in the absence of N-WASP, we immunostained paraffin embedded skin sections (P23, N-WASP^K14KO^ and control mice) for cytokeratin 10 (early differentiation) (Fig. S3A), involucrin (intermediate differentiation) (Fig. S3B), transglutaminase (terminal differentiation) (Fig. S3C) and cytokeratin K14 (Fig. S3D). Both intermediate and terminal differentiation proteins levels were increased in N-WASP^K14KO^ mice on P23 day (n = 3). Western blot analysis also showed similar results (Fig. S3E). In conclusion, increased numbers of BrdU and PCNA positive cells suggest that increased keratinocyte proliferation in the epidermis of N-WASP^K14KO^ mice leads to epidermal hyperplasia. Thus the N-WASP^K14KO^ mouse recapitulates key features of atopic dermatitis in human patients.

### N-WASP deficiency does not affect TPA induced hyperproliferation response

**Figure 3:**
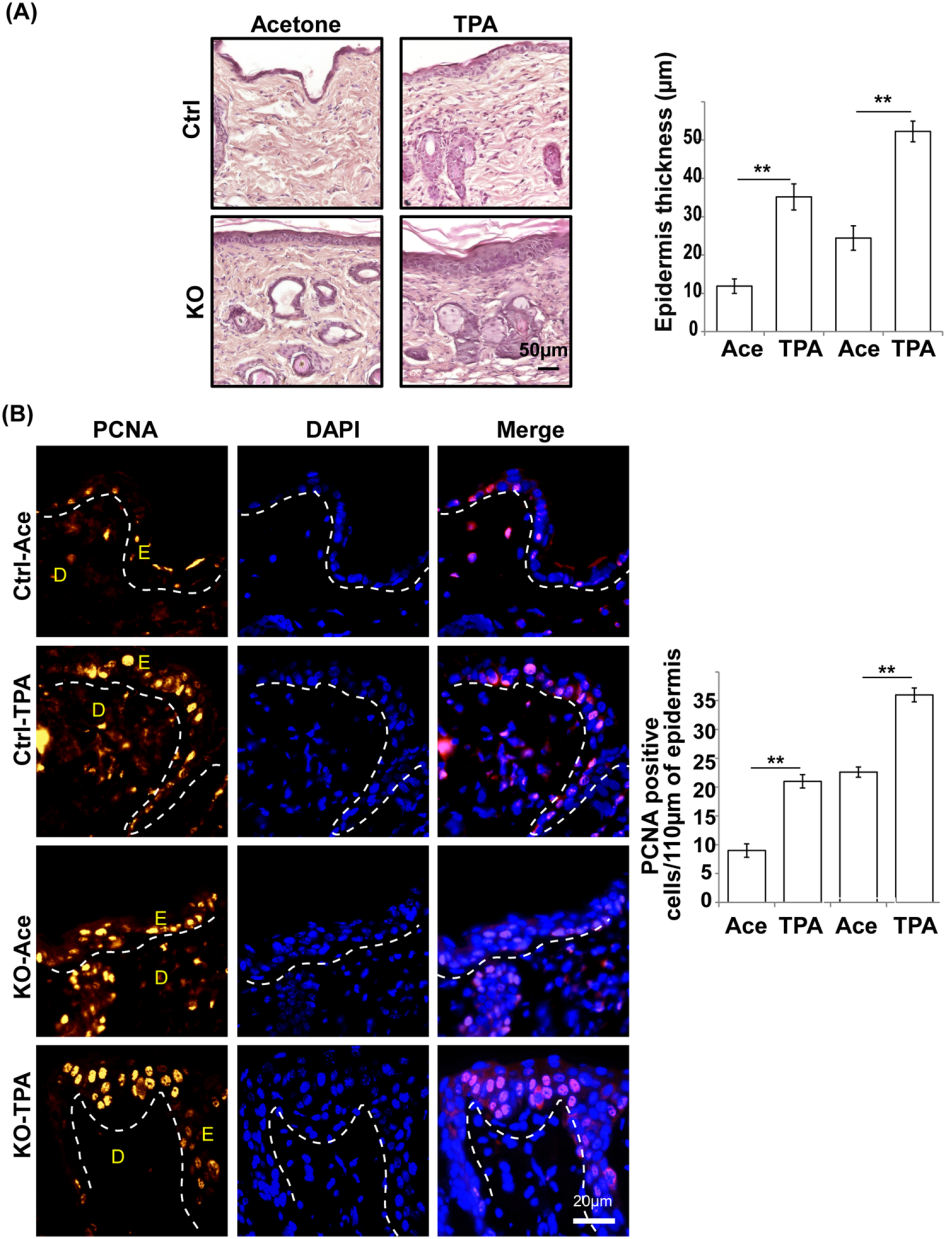
TPA-induced hyperproliferation in N-WASP^K14KO^ mice. **(A)** H & E staining of dorsal skin of control and N-WASP^K14KO^ mice 24 hours following application of solvent (acetone) or TPA. **(B)** Immunostaining and quantification of PCNA-positive cells in TPA treated skin from control and N-WASP^K14KO^ mice. (Ace: Acetone) Results are mean ± SEM ***p < 0.001, **p < 0.01, (n = 3).

N-WASP^K14KO^ mice have a thicker epidermis due to hyperproliferation of keratinocytes. To investigate whether the thicker epidermis leads to any altered cutaneous response, we carried out TPA induced inflammation assay (n = 3). We applied TPA (6.5 ng/ml) in acetone or acetone (TPA control) topically on the dorsal skin of control and N-WASP^K14KO^ mice and analyzed the skin by H&E staining 24 hours after treatment. TPA-treated control and N-WASP^K14KO^ mice displayed significant epidermal thickening compared to control and N-WASP^K14KO^ mice treated with vehicle (Fig. 3A). Histological revealed that TPA-treated skin of N-WASP^K14KO^ mice is much thicker compared to control mice (Fig. 3A). Corroborating this result, PCNA immunostaining also showed higher numbers of PCNA-positive proliferating keratinocytes in N-WASP^K14KO^ mouse skin (Fig. 3B). No significant change in epidermal thickening ratio (TPA/Vehicle, 2.62 WT, and 2.26 KO) was observed between the N-WASP^K14KO^ and control mice (Fig. 3A). This indicated that the response of N-WASP^K14KO^ epidermis to epidermal stress chemicals was similar to that of control mice. Immunofluorescence staining of TPA treated showed that there is no substantial difference in the E-cadherin expression pattern in both N-WASP^K14KO^ and controls (data not shown). These results imply that there is no defect in the epidermal cell-cell junction 24 hours following TPA application in both N-WASP^K14KO^ and control mice.

### Conditional keratinocyte-specific N-WASP knockout causes epidermal barrier function defects

**Figure 4:**
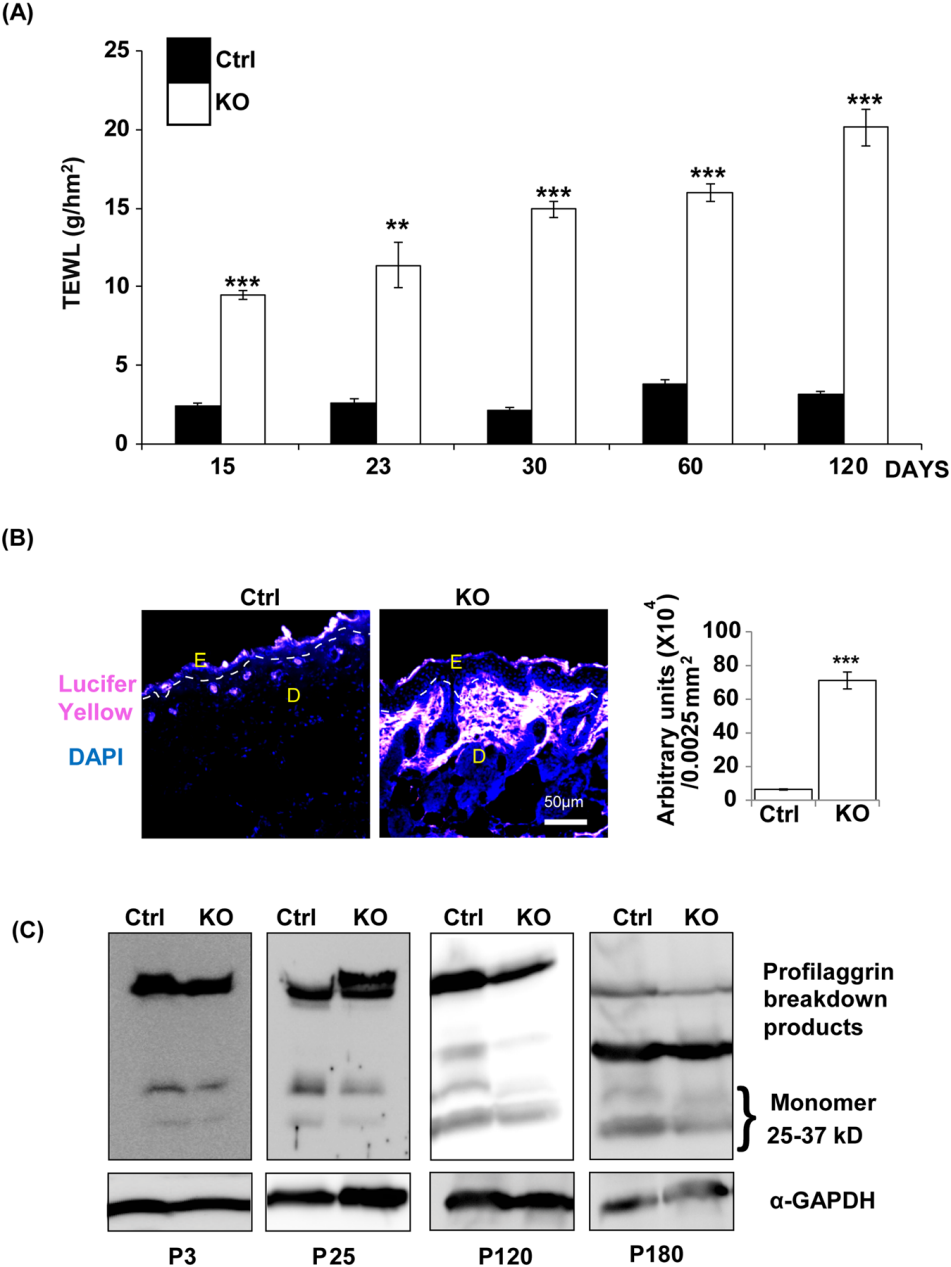
N-WASP knockout in keratinocytes causes a defect in epidermal barrier function. **(A)** Tewameter analysis showed increased transepidermal water loss (TEWL) in N-WASP^K14KO^ mice skin compared to control mice on P15, P23, P30, P60 and P120 day (n = 6). **(B)** Enhanced penetration of Lucifer Yellow into the skin of **N-**WASP^K14KO^ mice compared to skin of control mice. **(C)** Western blot analysis showed comparably reduced filaggrin expression in N-WASP^K14KO^ mice skin compared to control mice on P3, P25, P120 and P180 (n = 3). Results are mean ± SEM ***p < 0.001, **p < 0.01.

The N-WASP^K14KO^ mice had very dry skin suggesting increased water loss in these mice compared to the control (Fig. 1D-I). In order to characterize epidermal barrier function, we analyzed transepidermal water loss (TEWL) (n = 6) in control and N-WASP^K14KO^ on postnatal days 15, 23, 30, 60 and 120 using a Tewameter placed on the dorsal skin (Fig. 4A). Epidermal water loss in N-WASP^K14KO^ mice was significantly higher compared to control littermates at all-time points tested. TEWL was 2 fold higher in N-WASP^K14KO^ mice compared to control mice at P15 day and 6–8 fold higher at P120 day (Fig. 4A). After P120 days, TEWL of non-inflamed N-WASP^K14KO^ mice skin was also found to be 4 fold higher than controls (data not shown). The skin barrier defect in N-WASP^K14KO^ mice was further confirmed by Lucifer Yellow penetration assay (n = 3) which showed greater penetration in the skin of N-WASP^K14KO^ compared to controls (Fig. 4B).

Filaggrin plays an essential role in maintaining the epidermal barrier^33^. We thus analyzed the expression of filaggrin by western blot analysis which revealed a reduction in the skin of N-WASP^K14KO^ mice compared to controls at all the time points (P3, P25, P120 and P180 days) tested (Fig. 4C). In conclusion, increased TEWL and increased Lucifer Yellow penetration in N-WASP^K14KO^ mice suggests that conditional knockout of N-WASP in keratinocytes leads to reduced filaggrin levels and impaired epidermal barrier function.

### Expression of tight junction protein, Claudin-5 is reduced in N-WASP^K14KO^ mice

**Figure 5:**
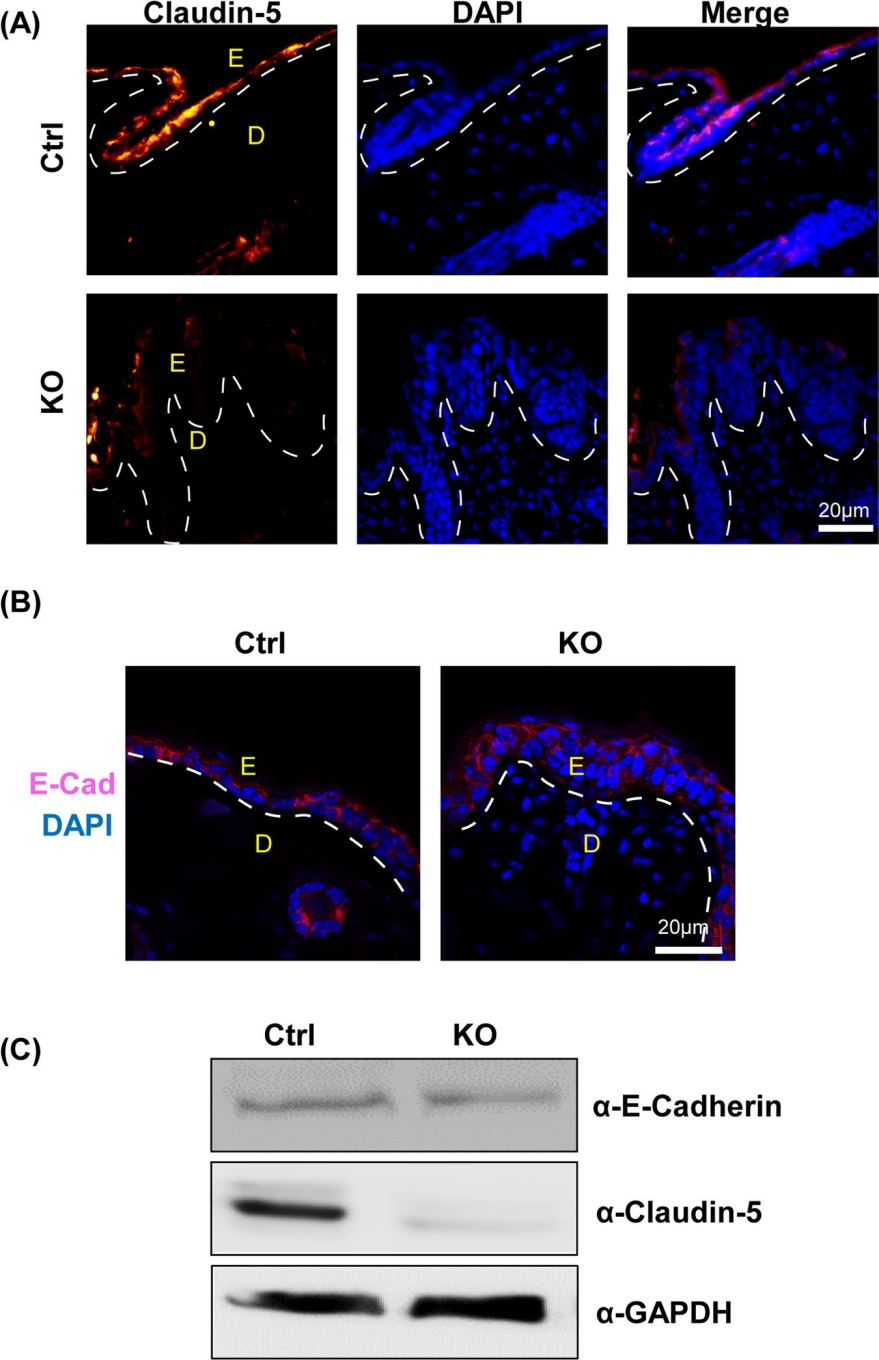
Expression of tight junction protein Claudin-5 is reduced in N-WASP^K14KO^ mice. Immunostaining of Claudin-5 **(A)** (n = 3) and E-cadherin **(B)** (n = 3) in N-WASP^K14KO^ and control mice skin showed reduced localization of Claudin-5 in N-WASP^K14KO^ but no significant change in E-cadherin localization. **(C)** Western blot analysis showed reduced Claudin-5 expression but no significant reduction in E-cadherin expression in N-WASP^K14KO^ compared to control mice skin (n = 3) (Blots were cropped and full length western blot images are in supplementary Fig. S6B).

The tight linkage of epithelial cells through adherens junctions, desmosomes and tight junction proteins contributes to the skin barrier function^34^. The crosslinking of structural proteins such as envoplakin, periplakin, and involucrin by transglutaminase forms the basis for the barrier in the form of corneodesmosomes. The subsequent conjugation of lipids, nuclear fragmentation and cell lysis ensure barrier function^35^. The increased water loss observed in N-WASP^K14KO^ mice could be due to defects in the cell-cell adhesion. To characterize the integrity of adherens and tight junctions, IF (immunofluorescence) was carried out for Claudin-5 (Fig. 5A) (n = 3), and E-cadherin (Fig. 5B) (n = 3) in P23 N-WASP^K14KO^ and control mice. IF staining revealed reduced localization of Claudin-5 in the epidermal layer of N-WASP^K14KO^ mice compared to control mice, but there was no observable difference in the localization of E-cadherin. Western blot analysis revealed a significant reduction of Claudin-5 expression in N-WASP^K14KO^ compared to controls (Fig. 5C), while the expression of E-cadherin was similar in both. Consistent with our results, the expression of Claudin-5 is decreased in the skin of atopic dermatitis mice^36^. In conclusion, ablation of N-WASP expression in keratinocytes led to the reduced expression and localization of tight junction proteins but not adherens junction proteins.

### Increased infiltration of immune cells in N-WASP^K14KO^ mice skin

**Figure 6:**
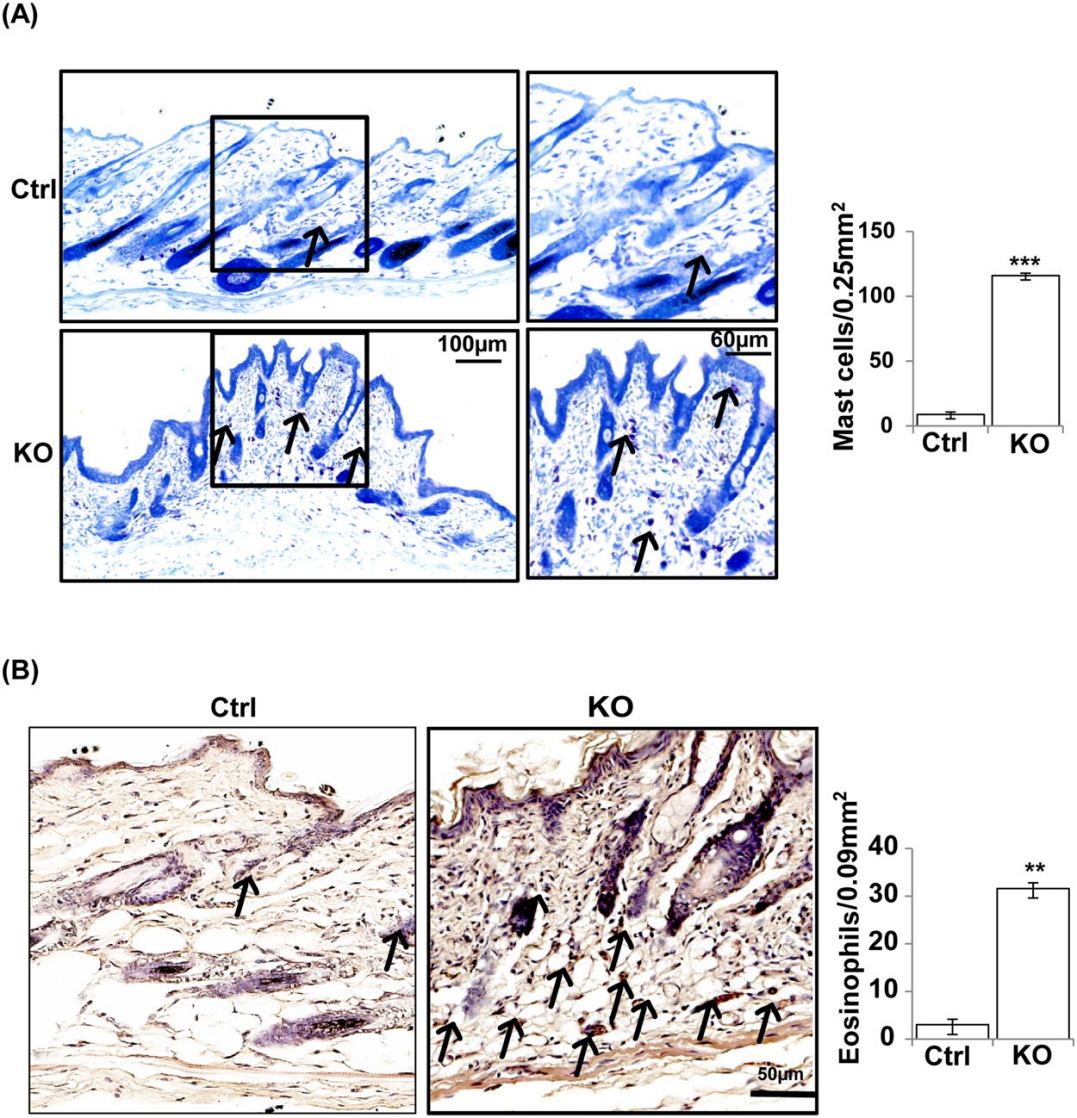
Increased mast cells and eosinophil cells in the skin of N-WASP^K14KO^ mice. **(A)** Toluidine blue staining showed significant increased infiltration of mast cells in dermis of skin in N-WASP^K14KO^ mice compared to control mice on 23^rd^ day. **(B)** Congo red staining showed significant increased infiltration of eosinophils in the dermal layer of N-WASP^K14KO^ mice compared to control mice on 23^rd^ day (n = 3). (↑) indicates mast cells and eosinophils. Results are mean ± SEM ***p < 0.001, **p < 0.01. (n = 3).

**Figure 7:**
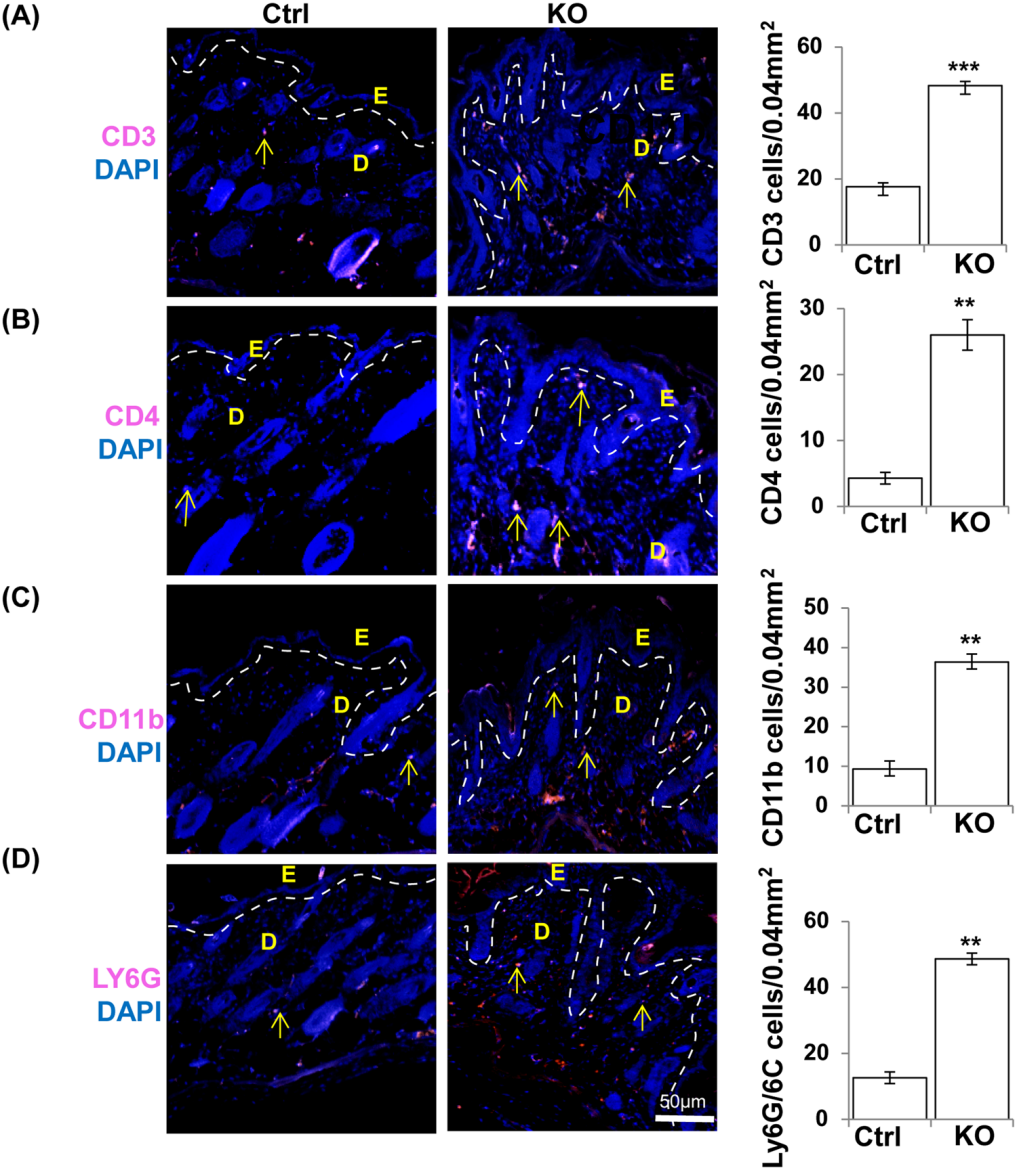
Increased CD3, CD4, CD11b and Ly6G/ly6C cells in the skin of N-WASP^K14KO^ mice. Immunostaining showed that CD3 **(A)**, CD4 **(B)**, CD11b **(C)** and Ly6G/ly6C **(D)** immune cells are significantly increased in dermal skin of N-WASP^K14KO^ mice compared to control mice on 23^rd^ day. (↑) indicates CD3, CD4, CD11b and Ly6G/ly6C cells. Results are mean ± SEM ***p < 0.001, **p < 0.01. (n = 3).

Defective barrier function of the epidermal layer of the skin leads to increased immune cell infiltration which is a clinical feature of acute atopic dermatitis^35^. Mast cells and eosinophils are more abundant in the skin of atopic dermatitis patients^25, 37^. To characterize immune cell infiltration, congo red (n = 3), toluidine blue (n = 3) and IF staining (n = 3) were carried out on N-WASP^K14KO^ and control mice at P19 and P23. Toluidine blue and congo red staining showed that infiltration of the skin by immune cells such as mast cells (Toluidine blue) and eosinophils (Congo red) was increased in N-WASP^K14KO^ mice compared to controls. Mast cell infiltration was significantly higher in the dermis of N-WASP^K14KO^ mice compared to controls (Fig. 6A, S4A). Mast cells are the major immune cells activated in skin inflammatory disease such as atopic dermatitis, allergic and eczema^37^. Eosinophils were recruited to the site of inflammation and predominantly distributed throughout the N-WASP^K14KO^ mice skin compared to controls (Fig. 6B). In addition, IF staining of skin sections showed that CD3 (Fig. 7A, S4A) and CD4 (Fig. 7B, S4C) positive lymphocytes were increased more than 2 fold in N-WASP^K14KO^ compared to control mice. Th1 lymphocytes produce IL-2 and TNF-α which are responsible for cell-mediated immunity through activation of phagocytic cells, while Th2 lymphocytes produce IL-6, which is responsible for Th2 and Th-17 cells differentiation in ovalbumin-induced AD mouse skin^38^.

Similarly IF staining of CD11b (3 fold increase) for monocytes (Fig. 7C, S4D) and ly6G/ly6C (3.2 fold increase) for neutrophils (Fig. 7D S4E) showed significant increases in N-WASP^K14KO^ compared to control mice. These results suggest that, in N-WASP^K14KO^ mice, increased infiltration of Th2 lymphocytes may activate and recruit mast cells and eosinophils to the dermis. In conclusion, Th-1 and Th-2 immune cells produce or stimulate the production of pro-inflammatory and proliferative cytokines and chemokines responsible for an exaggerated immune response against allergens or cutaneous lesions in N-WASP^K14KO^ mice.

### Development of local and systemic inflammatory responses in N-WASP^K14KO^ mice

**Figure 8:**
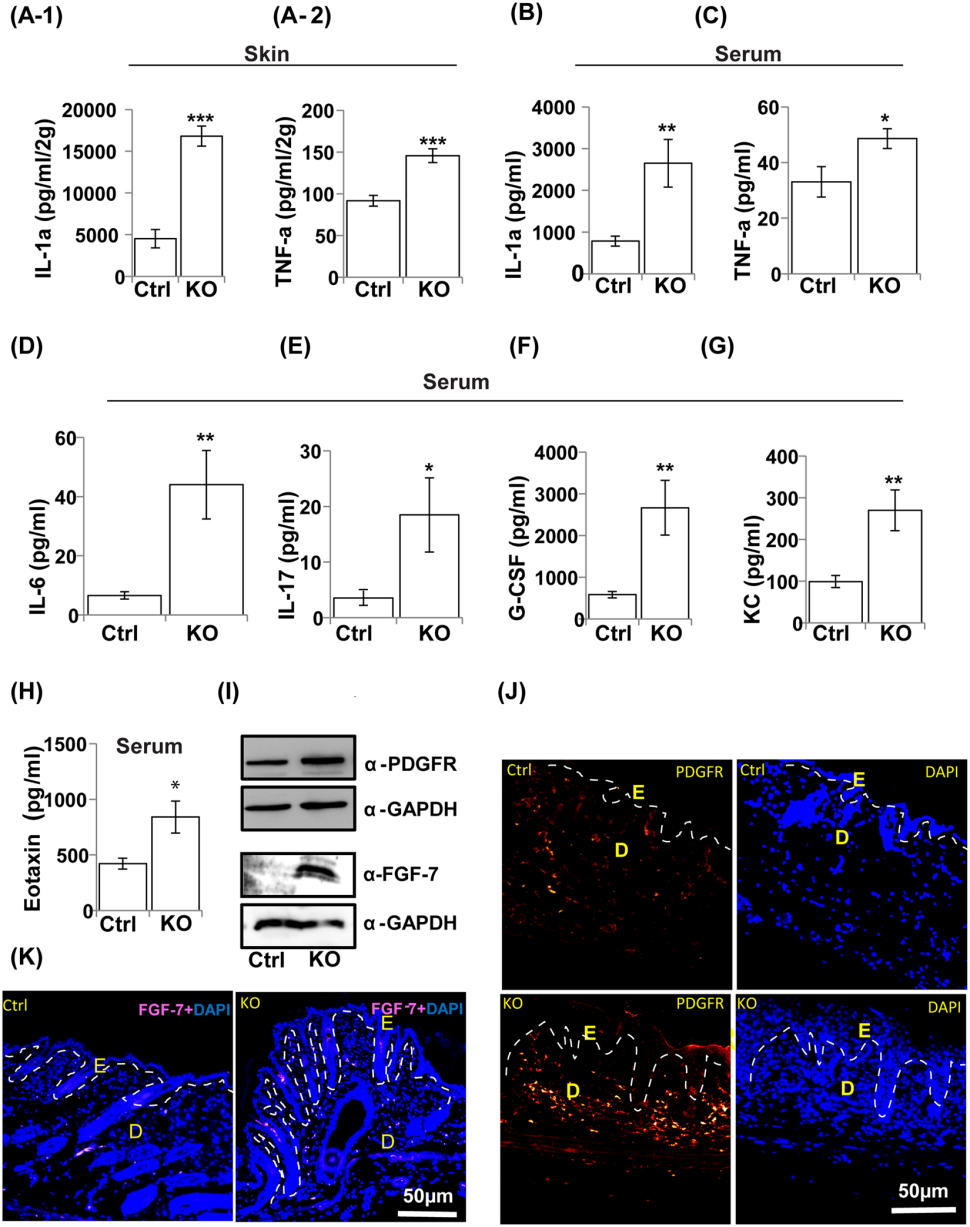
Development of local and systemic inflammatory responses in N-WASP^K14KO^ mice. Cytokine analysis showed higher levels of IL-1α **(A-1)** and TNF-a **(A-2)** in skin and as well as in serum **(B** and **C)**, IL-6 **(D)** and IL-17 **(E)** cytokines in serum of N-WASP^K14KO^ compared to control mice on 23^rd^ day. Similarly, chemokine analysis showed significantly increased levels of G-CSF **(F)**, KC **(G)** and eotaxin **(H)** chemokines in serum of N-WASP^K14KO^ mice compared to control mice on 23^rd^ day. **(I)** Western blot analysis of whole skin protein lysate showed that expression of PDGFR and FGF-7 are increased in N-WASP^K14KO^ compared to P23 control mice (Blots were cropped and full length western blot images are in supplementary Fig. S6C). Immunostaining showed that both PDGFR **(J)** and FGF-7 **(K)** are significantly increased in skin of N-WASP^K14KO^ mice compared to control mice (P23 mice). Results are mean ± SEM ***p < 0.001, **p < 0.01, *p < 0.05 (n = 8).

Systemic immune responses are commonly produced in atopic dermatitis patients by increased serum IgE, Th2 cytokines and chemokines and peripheral blood eosinophilia^39^. To characterize the development of systemic immune responses in N-WASP^K14KO^ mice, we collected blood at P23 (N-WASP^K14KO^ mice (n = 8) and control mice (n = 8)) and used the serum to determine the cytokine and chemokine profiles. Cytokines including Interleukin-1α (IL-1α in skin and serum) (Fig. 8A-1 and B), Tumor necrosis factor-α (TNF-α in skin and serum) (Fig. 8A-2 and C)), Interleukin-6 (IL-6) (Fig. 8D) and Interleukin-17 (IL-17) (Fig. 8E) were found to be significantly higher in the serum of N-WASP^K14KO^ mice compared to control mice. IL-1α and TNF-α are the major proinflammatory cytokines secreted by immune cells and keratinocytes. Interestingly, IL-6 cross talks with IL-1α and TNF-α to create an inflammatory response in cutaneous inflammation of the skin^27^. The level of IL-17 in the serum of N-WASP^K14KO^ was 5.2 fold higher compared to control mice (Fig. 8E). IL-17 is an inflammatory cytokine produced by lymphocytes and keratinocytes and is significantly increased in atopic dermatitis patients^40^.

Chemokines such as granulocyte-colony stimulating factor (G-CSF) (Fig. 8F), keratinocyte chemoattractant (KC) (Fig. 8G) and eotaxin (Fig. 8H) were increased significantly in the serum of N-WASP^K14KO^ mice compared to control mice. G-CSF is produced by keratinocytes, fibroblasts and endothelial cells, and is essential for neutrophil-dependent phagocytosis and neutrophil infiltration^41^. Eotaxin plays a crucial role in recruiting eosinophils to the site of skin inflammation and produces a strong allergic inflammatory response in atopic dermatitis. Monocytes and macrophages stimulate the secretion of keratinocyte chemoattractants (KC, IL-8) in skin lesions which function as chemoattractants for neutrophil accumulation^42^. These results suggest that IL-1α and TNF-α are the proinflammatory cytokines increased in cutaneous inflammation in N-WASP^K14KO^ mice which subsequently activate immune cells to produce IL-6, IL-17 and chemokines (G-CSF, Eotaxin and KC) leading to a systemic immune response against skin lesion.

### N-WASP knockout in keratinocytes leads to increased expression of PDGFR and FGF-7

Fibroblast proliferation and activation are increased in atopic dermatitis patients skin samples, as well in IL-13-induced atopic dermatitis mice^43^. Fibroblasts secrete cytokines, growth factors and keratinocyte mitogen which are involved in keratinocyte proliferation in epidermis^43^. To characterize fibroblast proliferation and secretion of keratinocyte mitogen from dermal fibroblasts in P23 N-WASP^K14KO^ mice (n = 3) and controls (n = 3), IF staining and western blot (whole skin samples) were carried out for the fibroblast marker PDGFR and FGF-7 (keratinocyte growth factor, KGF). Western blot results revealed that the expression of PDGFR and FGF-7 were increased significantly in N-WASP^K14KO^ mice compared to controls (Fig. 8I). In addition, IF staining also showed that PDGFR (Fig. 8J) and FGF-7 (Fig. 8K) were increased in P23 N-WASP^K14KO^ mice compared to P23 control mice. PDGF and PDGFR have been implicated in the control of dermal thickness by regulating fibroblast proliferation, migration and survival^44^. FGF-7 is a keratinocyte mitogen^45^ produced by fibroblasts activated by cytokines such as IL-1α and TNF-α secreted from epidermal cells^45^. In conclusion, elevated level of PDGFR expression and increased numbers of PDGFR positive cells suggest that N-WASP^K14KO^ mice have increased numbers of fibroblasts and this may be responsible for the increased secretion of FGF-7 and keratinocyte hyperproliferation.

## Discussion

N-WASP is a ubiquitously expressed WASP family protein which regulates actin polymerization by modulating the activity of the Arp2/3 complex^3^. N-WASP is expressed in the early stages of murine embryonic development and total N-WASP loss of function caused embryonic lethality at E11, suggesting an essential role in embryonic development^46^. Conditional knockout of N-WASP in muscle using Myf5-cre and MyoD-cre^47^ likewise lead to embryonic lethality while conditional knockout of N-WASP in brain using Nestin-cre^48^ was lethal postnatally. In previous studies, conditional knockout of N-WASP in keratinocytes using K5-cre caused alopecia with normal epidermal development^28^ or alopecia with hyperproliferation^29^. Both studies had focused on the role of N-WASP in hair follicle cycling^28, 29^. In the mice used by Lefever *et al*.^28^, the LoxP sites flanked exon 6–9 and, following Cre-mediated excision, and led to the expression of a truncated N-WASP peptide (exons1–5 and partial exon10) that was expressed in keratinocytes^28, 49^. In the mice used by Lyubimova *et al*.^29^, the LoxP sites flanked exon2 (135 nt), possibly leading to the splicing of exon1 to exon3 and expression of an internally deleted N-WASP peptide lacking the 45 amino acids encoded by exon2 in the keratinocytes of conditional knockout mice (Lyubimova 2010). The *N-WASP* ^*fl/fl*^ mice we generated have exons 3–4 flanked by LoxP sites and any splicing of exon2 to exon5 will cause premature termination of translation; only a peptide of 99 amino acids including the first 81 amino acids of N-WASP will be expressed. To further characterize the role of N-WASP in keratinocytes in skin development and homeostasis, we generated mice with conditional knockout of N-WASP in keratinocytes using K14-cre as the driver. N-WASP^K14KO^ mice were born at the expected Mendelian ratio suggesting that expression of N-WASP in keratinocytes is not essential for embryonic development.

The N-WASP^K14KO^ mice had growth defects as determined by body mass measurements and were smaller than control littermates at all time points analyzed. The reduced weight of the N-WASP^K14KO^ mice could be due to defects in the esophagus and/or increased TEWL^30, 31^. These mutant mice also exhibited dry and scaly skin attributable to an increased transepidermal water loss seen in the N-WASP^K14KO^ mice. Histological skin sections of N-WASP^K14KO^ mice showed a thicker epidermis compared to controls and a higher number of PCNA and BrdU positive cells suggesting that the keratinocytes in the epidermis of N-WASP^K14KO^ mice were undergoing hyperproliferation. It has been suggested that hyperproliferation and acanthosis are compensatory responses to defects in epidermal barrier function^50^. IF staining revealed a reduction inexpression and localization of Claudin-5 in N-WASP^K14KO^ mice compared to controls, and similarly N-WASP^K14KO^ mice had reduced expression of filaggrin relative to controls. The Lucifer Yellow penetration assay also revealed impaired epidermal barrier function in N-WASP^K14KO^ mice. Thus, the increased transepidermal water loss in N-WASP^K14KO^ mice is probably due to the impaired epidermal barrier caused by deficiency in expression and localization of tight junction protein (Claudin-5) and reduced expression of filaggrin. Tight junction proteins help to maintain epidermal barrier, thus suggesting that N-WASP deficiency in our animal model led to a reduction in expression of filaggrin and Claudin-5. Previous reports have also mentioned that mutation in filaggrin or filaggrin deficiency leads to decreased stratum corneum hydration as a result of increased TEWL, which correlates with results obtained from human AD patients^51^. It is also possible that epidermal barrier defects caused an infiltration of immune cells that caused the atopic dermatitis.

Epidermal immune cells and keratinocytes play a crucial role in the immune responses in inflammatory skin diseases, such as atopic dermatitis and psoriasis^35^. Firstly, N-WASP^K14KO^ mice had dry wrinkled scaly skin, inflammation on the neck and face (n = 20), and there were nevi on the dorsal skin of P180 mice. N-WASP^K14KO^ mice had severe alopecia after P120 which is one of the primary clinical features in atopic dermatitis patients^35^. Secondly, the skin of N-WASP^K14KO^ mice had an epidermal barrier defect due to reductions in filaggrin and the tight junction protein Claudin-5. Both of these attributes are similar to that seen in human atopic dermatitis patients. Thirdly, N-WASP^K14KO^ mice exhibited epidermal thickening which is characterized by elevated epidermal keratinocyte proliferation as observed in AD skin samples^52^. Fourthly, high levels of mast cells and eosinophil infiltration in the skin of N-WASP^K14KO^ mice, are consistent with features of the skin of atopic dermatitis and psoriasis patients. Finally, N-WASP^K14KO^ mouse serum exhibited higher levels of proinflammatory cytokines and chemokines, including IL-1α (serum and skin), TNF-α (serum and skin), IL-6, IL-17, G-CSF and eotaxin. All these factors are also involved in the initiation of pathogenesis in AD patients^53^. They are likewise implicated in the systemic immune response in N-WASP^K14KO^ mice which is similar to that seen in AD patients^54^. In order to determine the role of N-WASP in AD pathogenesis in humans, we analysed the expression of N-WASP in AD patients using published microarray data^55^. We found that the expression of N-WASP was reduced by 50% in AD patients (n = 10) compared to healthy individuals (n = 10) (Fig. S5), however the difference was not statistically significant probably because of biological variance in the expression of N-WASP and the small number of subjects.

Accumulation of immune cells in the dermis of AD patient skin is a clinical hallmark of atopic dermatitis. In the present study, we found increased infiltration of allergic immune cells such as mast cells (10 fold) and eosinophils in the dermis of mutant mouse skin. Recent studies have shown that proliferation, migration, and local activation of mast cells^37^ and eosinophils are the characteristic features of AD^35^. IF staining also revealed increased infiltration of CD3, CD4 (2 fold), Ly6G/Ly6C (3.2 fold) and CD11b (3 fold) positive cells in N-WASP^K14KO^ skin (Fig. 7, S4). Our results are consistent with reports stating that different T lymphocytes (Th1 and Th2 cells) infiltrate at the site of skin lesion and secrete cytokines and chemokines to maintain the pathology of atopic dermatitis^56^. The initiation and development of atopic dermatitis is organised by the local tissue expression of proinflammatory cytokines and chemokines. Cytokines such as TNF-α and IL-1 from keratinocytes, mast cells and dendritic cells induce the expression of adhesion molecules on vascular endothelial cell which facilitates the extravasation of inflammatory cells into the skin^57^. We found that the levels of both TNF-α and IL-1α are significantly elevated in the serum of N-WASP^K14KO^ mice compared to control mice. These findings suggest that proinflammatory cytokines play a major role in the recruitment of more immune cells and maintain the pathogenesis of AD in N-WASP^K14KO^ mice skin. It has also been reported that the ratio of IL-1α and the IL-1α receptor are significantly increased in patient samples with cutaneous inflammation such as atopic dermatitis and psoriasis^58^. Moreover, in our study, we found that the Th1 and Th2 cytokines and chemokines such as IL-6, G-CSF, eotaxin, KC and IL-17 were significantly elevated in the serum of N-WASP^K14KO^ mice. These results suggest that Th2 lymphocytes activated by proinflammatory cytokines (IL-1α) induce the secretion of Th2 cytokines (IL-6 and IL-17) to produce systemic and humoral immune responses in atopic dermatitis-like lesions of N-WASP^K14KO^ mice. Thus, our results are similar to those of several previous results which showed that highly elevated levels of proinflammatory cytokines promote the differentiation of Th0 lymphocyte cells to Th2 cells, leading to elevated levels of Th2 cytokines and chemokines such as IL-4 and IL-13 as seen in acute atopic dermatitis^43^. Moreover, FGF-7 which is produced from dermal fibroblast was increased in N-WASP^K14KO^ mouse skin. Several reports have stated that FGF-7 plays a critical role in regulating epidermal keratinocyte proliferation and both KGF (FGF-7) and FGF10 have been shown to be increased in paraffin sections of psoriasis patient skin samples^59^. Notably, PDGFR expression was also increased in N-WASP^K14KO^ mice suggesting an increased presence of dermal fibroblasts.

In summary, our results suggest that conditional knockout of N-WASP in keratinocytes leads to AD-like inflammation and a systemic immune response in the skin. Ablation of N-WASP expression increased epidermal water loss is due to skin barrier defects which could be due to the reduced levels of Claudin-5 and filaggrin which play vital roles in maintaining epidermal barrier function. This leads to dry skin, activation of immune cells and keratinocytes in the epidermis, and production of proinflammatory cytokines which induce dermal T lymphocytes to produce IL-6 and IL-17 inflammatory cytokines. Subsequently, other dermal immune cells such as neutrophils, leukocytes, mast cells and eosinophils are recruited to the site of skin inflammation. On the other hand, proinflammatory cytokine (IL-1α) activates fibroblasts to produce keratinocyte mitogen (FGF-7) which enhances keratinocyte proliferation. These results suggest that N-WASP is essential for maintaining the skin barrier and skin homeostasis in adult mice skin. We have identified a novel role for N-WASP in the etiology of AD and our results suggest that N-WASP^K14KO^ adult mice can be used as a model to study human AD.

## Materials and Methods

### Animals

Keratinocyte specific N-WASP knockout mice were generated by crossing *N-WASP* ^*fl/fl*^ female mice^48^ with male K14-cre mice (keratinocyte specific protein, Jackson Laboratory Stock No. 018964). Heterozygous *N-WASP* ^*fl/WT*^ *K14-cre* mice obtained from the first generation were back crossed with *N-WASP* ^*fl/fl*^ to get *N-WASP* ^*fl/fl*^ *; K14-cre* (N-WASP^K14KO^) homozygous mice. Mice genotyping were performed as described^48^. Briefly, small pieces of tail tips (2–5 mm) were digested in tail digestion buffer containing 50 mM KCl, 10 mM Tris–HCl (pH 9.0), 0.1% Triton X-100, and 0.4 mg/mL Proteinase K (Sigma-Aldrich; P2308). Tail PCR was done using KAPA HiFi PCR Kit (KK2101). The primers used to detect flox/flox were (5′ to 3′ direction); AGCTCAGAGAAGGTGTATTGG (forward) and AGGACTTACATCTCCAGCAAAGG (reverse). Cre primers: TCCAGGGCGCGAGTTGATAG-3′ (forward), CGATGCAACGAGTGATGAGG (reverse). Mice were maintained at constant temperature of 20 °C with 12-hour light/12-hour dark cycle and were given a balanced diet with unlimited water. All the animal experiments carried out were approved by Institutional Animal Care and Use Committee (IACUC) of Nanyang Technological University (NTU), (ARF-SBS/NIE-A0237, ARF-SBS/NIE-A0250, and ARF-SBS/NIE-A0323). All the experiments were performed according to approved protocols of Institutional Animal Care and Use Committee.

### Transepidermal water loss (TEWL) measurement

N-WASP^K14KO^ and control mice models were used for transepidermal water loss measurement using the Tewameter TM210 with Multi Probe Adapter (CK electronic GmbH, Koln, Germany) as per manufacturer’s operating instructions. Data was expressed in g/m^2^h. (n = 6).

### Haematoxylin and Eosin staining

N-WASP^K14KO^ and control mice were anaesthetized and sacrificed by cervical dislocation. Skin samples from dorsal, ear and tail of mice were leveled on parafilm and fixed with 4% paraformaldehyde overnight at 4 °C. Fixed skin samples were washed with 1X PBS and then dehydrated by sequential transfer of the skin through ethanol solution (70, 80, 90 and 100%) for 1 hour each. After ethanol wash, the samples were treated with 50% xylene/ethanol mixture and then twice with 100% xylene solution. Dehydrated samples were then immersed in paraffin wax at 60 °C overnight and embedded in paraffin blocks. The embedded tissues were sectioned, 5 μm size on superfrost slides (Fisher). The slides were kept at 60 °C to remove the paraffin for staining and subsequently rehydrated and stained with haematoxylin and eosin.

### Toluidine blue and Congo red staining

Toluidine staining was performed by treating tissue sections with toluidine stain (0.5% toluidine blue solution in water) for 30 seconds. Congo red staining was performed by immersing tissue section in congo stain (1% congo red solution in water) for 5 minutes. The slides were washed with water and dipped in 2.5% KOH solution and subsequently counterstained with haematoxylin. Sections were washed in tap water and then dehydrated by increasing the concentration of alcohol (80, 90 and 100%), cleared and mounted in DPX. Slides were assessed in 20X and 40X magnification.

### Lucifer Yellow penetration assay

We placed 1 cm^2^ piece of dorsal skin of N-WASP^K14KO^ (n = 3) and control mice (n = 3) in a petri dish and added 20 ul of 1 mM of Lucifer Yellow, and incubated at 37 °C for 1 hr. After incubation the skin was washed with PBS and embedded in OTC and cryo-sectioned. Cryo-sections were stained with DAPI and analyzed with fluorescence microscope^60^.

### BrdU staining

Mice (N-WASP^K14KO^ (n = 4) and control mice (n = 4)) were injected intraperitoneally with BrDU (100 mg/kg). Dorsal skin biopsies were collected 24 hrs after BrdU injection and embedded in paraffin. Paraffin sections were used for BrdU immunofluorescent staining^61^.

### Immunofluorescence

IF staining was carried out on paraffin sections according to Jain *et al*.^48^. Paraffin sections were deparaffinized, rehydrated and treated with citrate buffer (pH6.0) at 95–100 °C for 20 min for antigen retrieval. Slides were blocked with 1% BSA for 1 hr and incubated with primary antibodies, overnight at 4 °C. Slides were then incubated with secondary antibodies conjugated with alexa fluor 594 for an hour at room temperature. Nuclei were visualized with DAPI staining for 30 min. After staining, the slides were washed with 1X PBS, subsequently dehydrated and mounted with DPX mounting media. IF images were captured using Olympus microscope with CoolSNAP^HQ2^ camera. All IF individual experiments were repeated thrice and data were analyzed and quantified using ImageJ software^62^. The dilution of the antibodies used were as follows: PCNA, K10 (Santa Cruz, 1:100), Involucrin (1:100,Santa Cruz), Filaggrin (Abcam, 1:100), CD11b (Santa Cruz 1:100), CD4 (Santa Cruz, 1:100), CD3 (Santa Cruz, 1:100), Ly6G/Ly6C (Biolegend, 1:100), Claudin-5 (Santa Cruz, 1:50), E-cadherin (BD biosciences, 1:100), BrdU (eBioscience, 1:50), K14 (1:10, LL001 from gift from Birgit’s lab), Alexa fluor 594 goat anti-rabbit (Molecular Probes Life Technologies, 1:500), Alexa fluor 594 goat anti mouse (Molecular Probes Life Technologies, 1:500).

### Cytokine array analysis

Mouse cytokine and chemokine array 31 plex was carried out on serum sample of control (8 mice) and N-WASP knockout mice (8 mice) in Eve technology (Canada). The serum samples were prepared according to the Eve technology protocol.

### ELISA

The level of IL-1α and TNF-α were analyzed in skin lysate of N-WASP^K14KO^ knockout mice (8 mice) and control mice (8 mice) using ELISA (eBioscience, CA92121USA).

### Quantification of *Staphylococcus aureus*

Skin swabs from the ear (1 cm^2^) of N-WASP^K14KO^ (n = 3) and control mice (n = 3) were placed in 1 mL of sterile PBS and diluted 10 fold with PBS. 50 µl of PBS bacterial suspensions were plated on mannitol salt medium and the plates were incubated for 2 days at 37 °C. Yellow color colonies that appeared were counted and expressed as log colony-forming units. The identity of the bacteria (*S. aureus*) was further confirmed by Gram staining, coagulase test and PCR using *S. aureus* enterotoxin B (SEB) specific Primers 5′-TCGCATCAAACTGACAAACG, 3′-GCAGGTACTCTATAAGTGCCTGC^63^.

### TPA induced hyperproliferation

TPA (6.5 nM/50 µl in acetone) was topically applied on the dorsal skin of control mice and N-WASP^K14KO^ mice. After 24 hrs of the treatment, mice were sacrificed and the skin sample was collected and fixed in 4% paraformaldehyde/PBS at 4 °C overnight and then embedded in paraffin for histological sections. Sections were stained with haematoxylin and eosin, PCNA (Santa Cruz, 1:100), Ly-6G/Ly-6C (Biolegend, 1:100) and E-cadherin (BD biosciences, 1:100) antibodies.

### Statistical analysis

Statistical significance analysis was performed using unpaired student’s t-test and *p* value <0.05 was considered as significant. Values in bar charts are the mean ± SEM from three independent experiments.

## Electronic supplementary material

Supplementary Information
