# Supplementary material for: Conditional knock out of N-WASP in keratinocytes causes skin barrier defects and atopic dermatitis-like inflammation
Source: Sci Rep. 2017 Aug 4;7:7311. doi: 10.1038/s41598-017-07125-8 (PMC5544743; doi:10.1038/s41598-017-07125-8)
Supplement: Supplementary file 1 — Supplementary Information [file 41598_2017_7125_MOESM1_ESM.pdf]

Conditional knock out of N-WASP in keratinocytes causes skin barrier defects and atopic dermatitis-like inflammation

+Pazhanichamy KALAILINGAM[1], + Hui Bing TAN[1], Neeraj JAIN[1], Ming Keat SNG[1,2], Jeremy Soon Kiat CHAN[1], Nguan Soon TAN[1,2,3,4] and Thirumaran THANABALU[1*]

Figure S1. Generation of N-WASP knockout mice in keratinocytes in skin

(A) Tail genomic PCR genotyping of 1st generation mice, **(B)** Tail genomic PCR genotyping of mice from second mating (*N-WASP*$^{fl/fl}$ X *N-WASP*$^{fl/WT}$*; K14-Cre*). Asterisk (*) indicates homozygous, *N-WASP*$^{fl/fl}$*; K14-Cre* mice, (↑) indicates heterozygous, *N-WASP*$^{fl/WT}$*; K14-Cre* mice. **(C)** Tail genomic PCR representing the deletion product of exon 3 and 4 of N-WASP gene. **(D)** H & E stained esophagus (n=5) sections of P23, N-WASP^{K14KO} and control mice **(E)** Bacterial colonies (*S. aureus*) obtained from ear swab samples of N-WASP^{K14KO} mice were higher compared to ear swab samples from control mice. **(F)** PCR amplification of *Staphylococcal* enterotoxin B (SEB) gene confirmed the identity of yellow colonies as *S. aureus* (n=3)**.** Results are mean ± SEM *** p<0.001, ** p<0.01

Figure S2. Loss of N-WASP expression in keratinocytes caused hyperproliferation of keratinocytes in epidermis of N-WASP^{K14KO} mice skin

(A) H & E stained dorsal skin (*N-WASP*$^{fl/fl}$), heterozygous (*N-WASP*$^{fl/WT}$*; K14-Cre*) and N-WASP^{K14KO} (*N-WASP*$^{fl/fl}$*; K14-Cre*), tail **(B)** and ear **(C)** sections of N-WASP^{K14KO} and control mice on 19th day (n=6). Quantification of epidermal thickening in skin **(D)**, ear (**E)** and tail (**F)** of control mice and N-WASP^{K14KO} mice skin sections (n=6). **(G)** PCNA immunostaining of paraffin embedded dorsal skin sections of control and N-WASP^{K14KO} mice (P19) and quantification of PCNA positive cells (n=3). Results are mean ± SEM *** p<0.001, ** p<0.01, *p<0.05

Figure S3. Ablation of N-WASP expression in keratinocytes does not affect differentiation of epidermis

Immunostaining of differentiation markers, cytokeratin 10 (early) **(A)**, involucrin (intermediate) **(B)**, transglutaminase (terminal) **(C)** and cytokeratin 14 **(D)** in paraffin embedded skin tissue of N-WASP^{K14KO} and control mice on P23 (n=3). Western blot analysis of expression of differentiation marker proteins **(E)** on P23 (n=3).

Figure S4. Increased infiltration of immune cells in N-WASP^{K14KO} mice skin

(A) Toluidine blue staining showed significant increased infiltration of mast cells in dermis of skin in N-WASP^{K14KO} mice compared to P19 control mice (n=3). Immunostaining for CD3 (n=3) **(B)** CD4 (n=3) **(C)** CD11b (n=3) **(D)** and Ly6G/6c (n=3) **(E)** showed a significant increase in immune cells in dermal layer of N-WASP^{K14KO} mice compared to control mice on 19th day**.** (↑) indicates mast cells, CD3, CD4**,** CD11b and Ly6G/ly6C cells. Results are mean ± SEM *** $p<0.001$, ** $p<0.01$.

Figure S5. Expression of N-WASP in healthy controls and atopic eczema patient samples

N-WASP expression levels in healthy controls and atopic eczema patients samples (The Data retrieved from GEO database (ID: GSE6012, Pubmed ID: 16918518, Probe ID for N-WASP: 205809_s_at) (n=10).

Figure S6. Full length western blot images of Fig. 1A, 5C and 8I

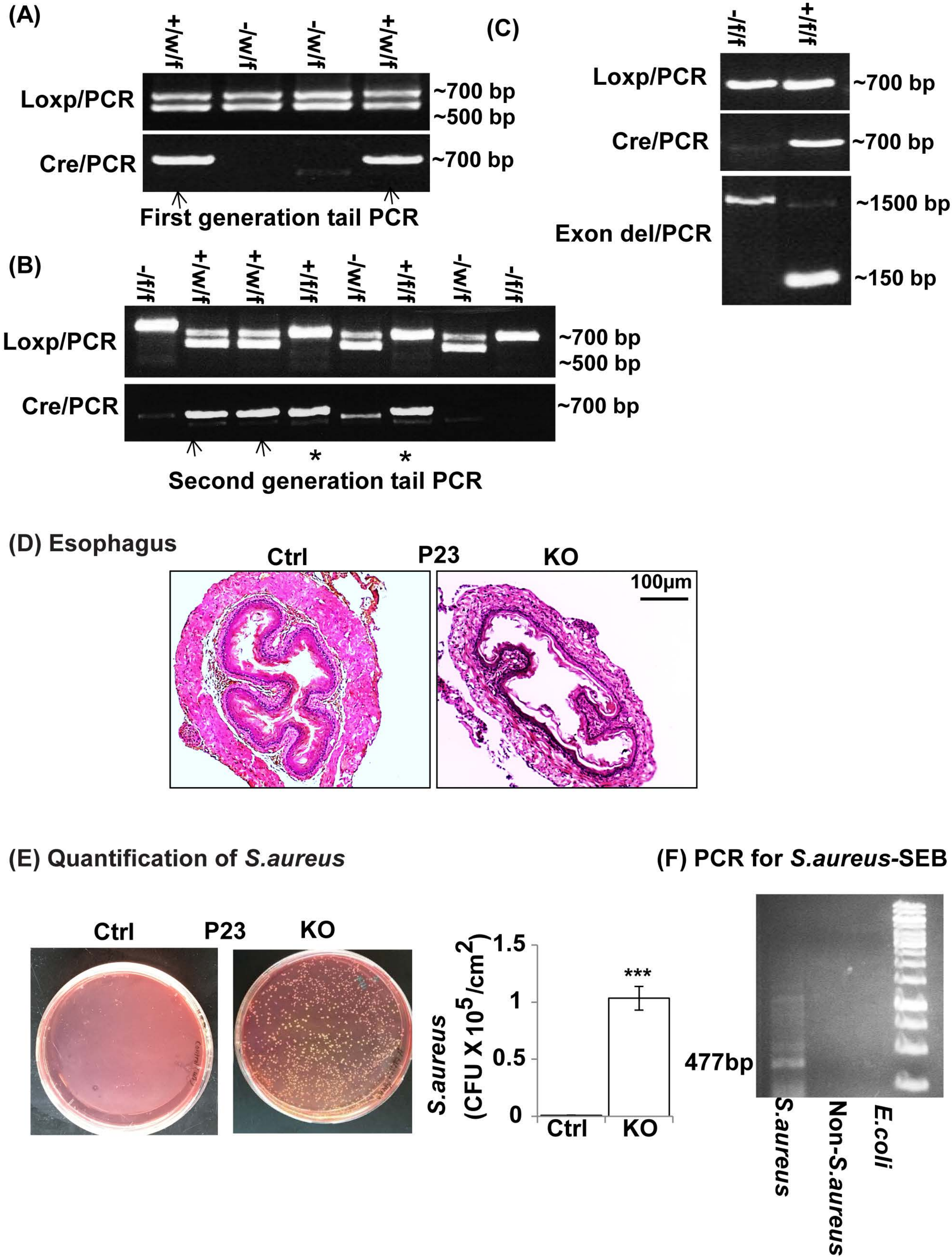

(A)
+/w/f
-/w/f
-/w/f
+/w/f
Loxp/PCR
~700 bp
~500 bp
Cre/PCR
~700 bp
First generation tail PCR
(B)
-/f/f
+/w/f
+/w/f
+/f/f
-/w/f
+/f/f
-/w/f
-/f/f
Loxp/PCR
~700 bp
~500 bp
Cre/PCR
~700 bp
*
*
Second generation tail PCR
(C)
-/f/f
+/f/f
Loxp/PCR
~700 bp
Cre/PCR
~700 bp
Exon del/PCR
~1500 bp
~150 bp
(D) Esophagus
Ctrl
P23
KO
100μm
(E) Quantification of S.aureus
Ctrl
P23
KO
S.aureus (CFU X 10^5/cm^2)
1.5
1
0.5
0
***
Ctrl
KO
(F) PCR for S.aureus-SEB
477bp
S.aureus
Non-S.aureus
E.coli

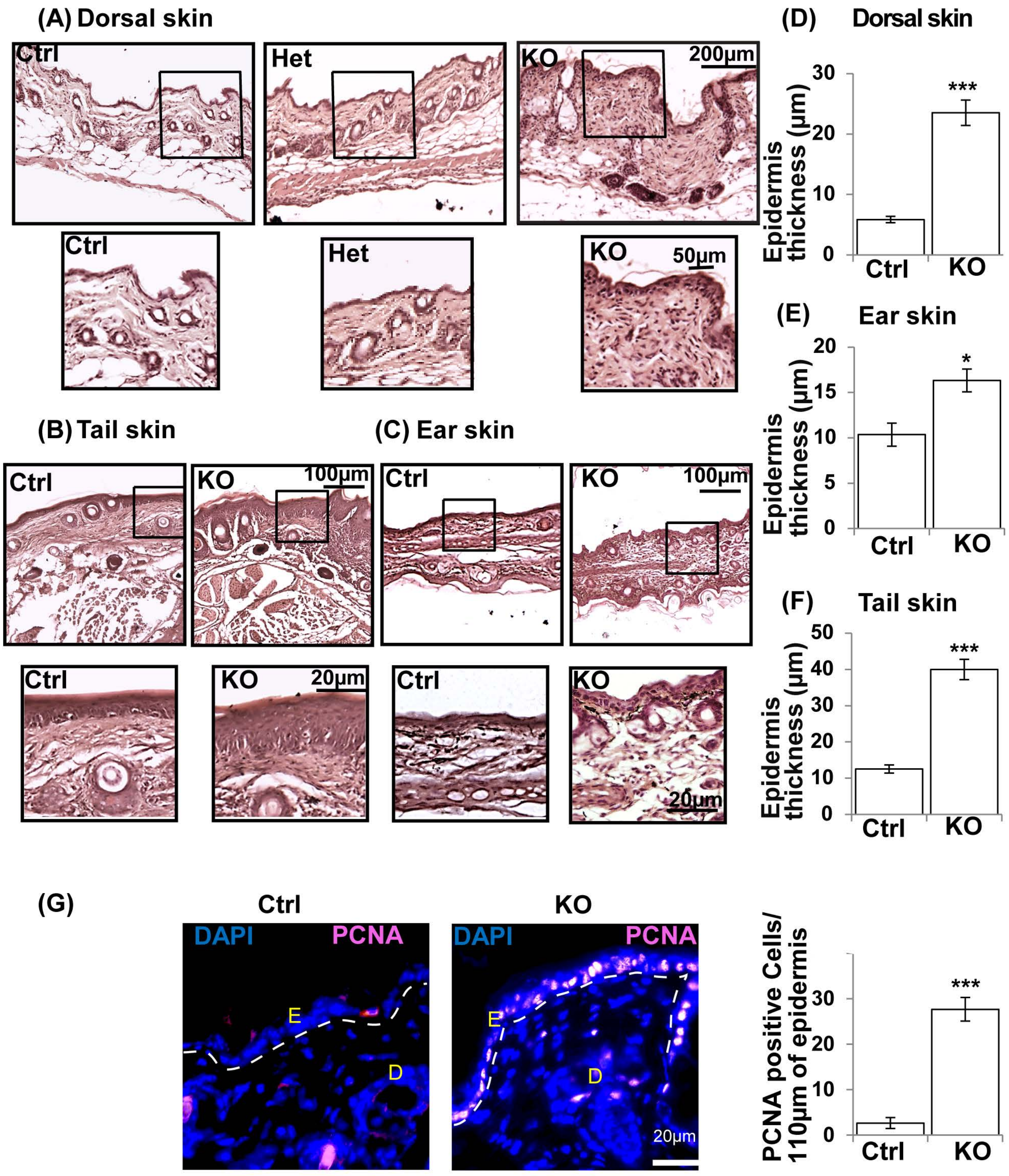

(A) Dorsal skin
Ctrl
Het
KO
200μm
Ctrl
Het
KO
50μm
(B) Tail skin
Ctrl
KO
100μm
Ctrl
KO
20μm
(C) Ear skin
Ctrl
KO
100μm
Ctrl
KO
20μm
(D) Dorsal skin
Epidermis thickness (μm)
0
10
20
30
***
Ctrl
KO
(E) Ear skin
Epidermis thickness (μm)
0
5
10
15
20
*
Ctrl
KO
(F) Tail skin
Epidermis thickness (μm)
0
10
20
30
40
50
***
Ctrl
KO
(G)
Ctrl
KO
DAPI
PCNA
E
D
20μm
PCNA positive Cells/ 110μm of epidermis
0
10
20
30
***
Ctrl
KO

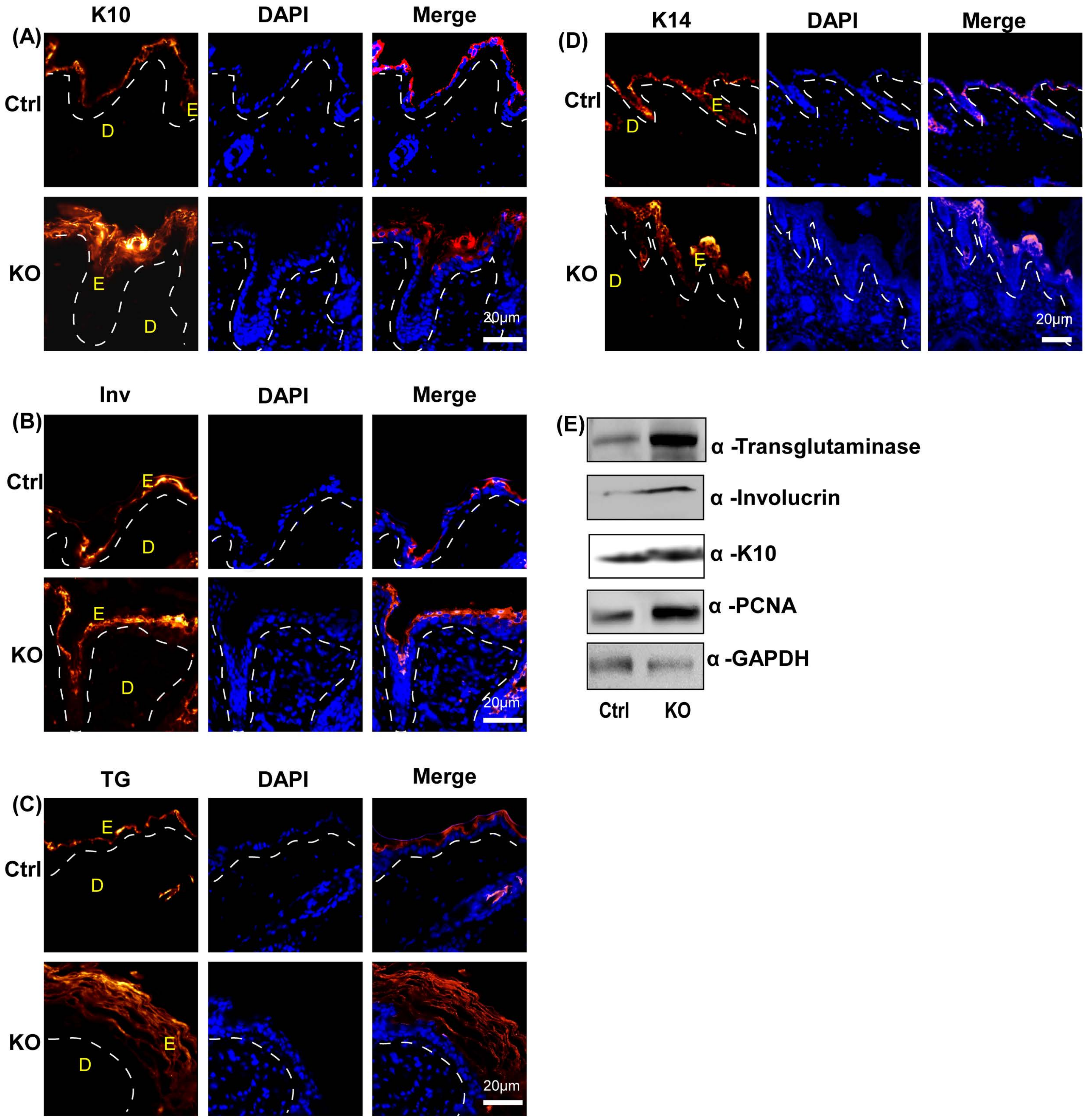

K10
DAPI
Merge
(A)
Ctrl
KO
E
D
20µm
Inv
DAPI
Merge
(B)
Ctrl
KO
20µm
TG
DAPI
Merge
(C)
Ctrl
KO
20µm
K14
DAPI
Merge
(D)
Ctrl
KO
20µm
(E)
α -Transglutaminase
α -Involucrin
α -K10
α -PCNA
α -GAPDH
Ctrl
KO

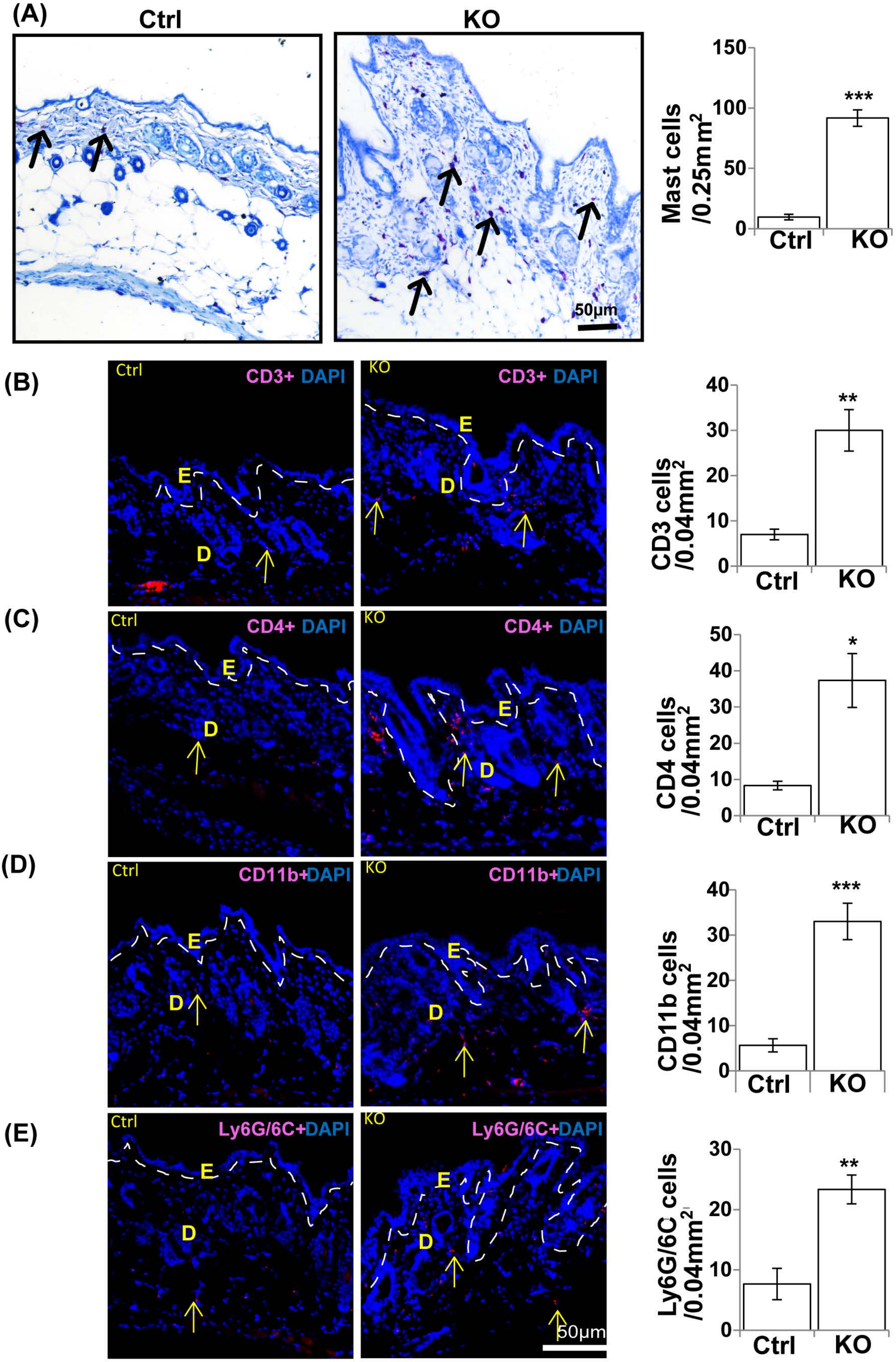

(A)
Ctrl
KO
50μm
Mast cells /0.25mm^2
150
100
50
0
***
Ctrl
KO
(B)
Ctrl
CD3+ DAPI
KO
CD3+ DAPI
E
D
CD3 cells /0.04mm^2
40
30
20
10
0
**
Ctrl
KO
(C)
Ctrl
CD4+ DAPI
KO
CD4+ DAPI
E
D
CD4 cells /0.04mm^2
50
40
30
20
10
0
*
Ctrl
KO
(D)
Ctrl
CD11b+DAPI
KO
CD11b+DAPI
E
D
CD11b cells /0.04mm^2
40
30
20
10
0
***
Ctrl
KO
(E)
Ctrl
Ly6G/6C+DAPI
KO
Ly6G/6C+DAPI
E
D
50μm
Ly6G/6C cells /0.04mm^2
30
20
10
0
**
Ctrl
KO

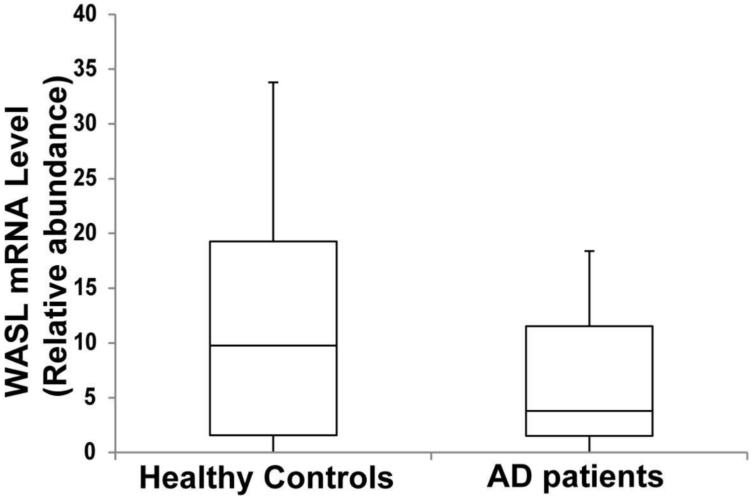

WASL mRNA Level
(Relative abundance)
40
35
30
25
20
15
10
5
0
Healthy Controls
AD patients

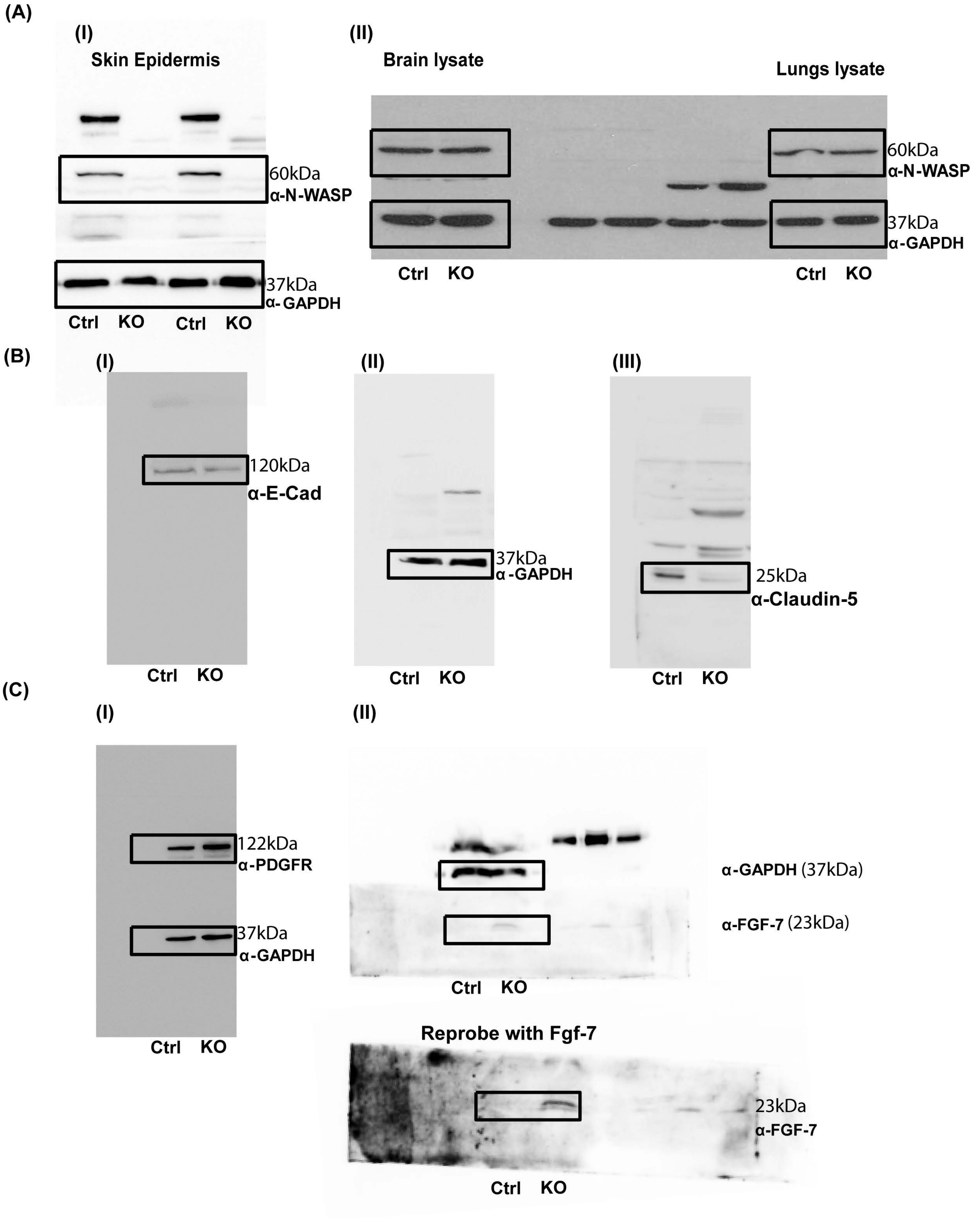
(A)
(I)
Skin Epidermis
60kDa
α-N-WASP
37kDa
α- GAPDH
Ctrl
KO
Ctrl
KO
(II)
Brain lysate
Lungs lysate
60kDa
α-N-WASP
37kDa
α-GAPDH
Ctrl
KO
Ctrl
KO
(B)
(I)
120kDa
α-E-Cad
Ctrl
KO
(II)
37kDa
α -GAPDH
Ctrl
KO
(III)
25kDa
α-Claudin-5
Ctrl
KO
(C)
(I)
122kDa
α-PDGFR
37kDa
α-GAPDH
Ctrl
KO
(II)
α-GAPDH (37kDa)
α-FGF-7 (23kDa)
Ctrl
KO
Reprobe with Fgf-7
23kDa
α-FGF-7
Ctrl
KO
